# Hydrogen migration in inner-shell ionized halogenated cyclic hydrocarbons

**DOI:** 10.1038/s41598-023-28694-x

**Published:** 2023-02-06

**Authors:** Abdul Rahman Abid, Surjendu Bhattacharyya, Anbu Selvam Venkatachalam, Shashank Pathak, Keyu Chen, Huynh Van Sa Lam, Kurtis Borne, Debadarshini Mishra, René C. Bilodeau, Ileana Dumitriu, Nora Berrah, Minna Patanen, Daniel Rolles

**Affiliations:** 1 J. R. Macdonald Laboratory, Department of Physics, Kansas State University, Manhattan, KS 66506 USA; 2 Nano and Molecular Systems Research Unit, University of Oulu, 90570 Oulu, Finland; 3 Department of Physics and Astronomy, Aarhus University, 8000 Aarhus, Denmark; 4 Department of Physics, University of Connecticut, Storrs, CT 06269 USA; 5 Hobart and William Smith Colleges, Geneva, NY 14456 USA

**Keywords:** Atomic and molecular interactions with photons, Atomic and molecular physics, Chemical physics

## Abstract

We have studied the fragmentation of the brominated cyclic hydrocarbons bromocyclo-propane, bromocyclo-butane, and bromocyclo-pentane upon Br(3*d*) and C(1*s*) inner-shell ionization using coincidence ion momentum imaging. We observe a substantial yield of CH_3_^+^ fragments, whose formation requires intramolecular hydrogen (or proton) migration, that increases with molecular size, which contrasts with prior observations of hydrogen migration in linear hydrocarbon molecules. Furthermore, by inspecting the fragment ion momentum correlations of three-body fragmentation channels, we conclude that CH_*x*_^+^ fragments (with *x* = 0, …, 3) with an increasing number of hydrogens are more likely to be produced via sequential fragmentation pathways. Overall trends in the molecular-size-dependence of the experimentally observed kinetic energy releases and fragment kinetic energies are explained with the help of classical Coulomb explosion simulations.

## Introduction

Although the fragmentation of multiply ionized polyatomic molecules (which may be induced, e.g., by photoionization, electron or proton impact, or strong-field ionization) primarily involves the *breaking* of molecular bonds, some fragmentation channels—typically occurring with relatively small yields—also involve the *formation* of new bonds. Intramolecular hydrogen or proton migration is one of the swiftest and most ubiquitous of such chemical changes^1–11^. It has been observed in a wide variety of molecular systems and was found to often occur on ultrafast timescales^1,7,8,12,13^.

Hydrogen (or proton) migration between DNA bases can trigger mutations^14^, which can eventually lead to cancerous cells. The migration can occur spontaneously, but extrinsic effects such as radiation or interaction with radicals can provoke similar dynamics leading to induced mutations^14^. In modern radiotherapy, induced mutations can be harnessed to kill cancerous cells very locally, for example, by initiating the damage using halogen radionuclides such as ^77^Br and ^125^I, which deposit energy to their immediate surroundings by electron emission through Auger-Meitner cascades^15^. Halogenated radiosensitizers such as halogenated nucleosides are also in clinical use, increasing the amount of DNA double-strand breaks, for example, via their enhanced cross-section for dissociative electron attachment^16^. In order to investigate the fundamental processes behind these complex sequences of events leading to mutations and radiation damage, such as hydrogen/proton transfer and fragmentation, studies on model gas-phase targets, especially using multi-coincidence techniques, are a valuable first step. Many experiments are reported in the literature that probe hydrogen migration in ionized gas-phase molecules. Some are performed using a single pulse, e.g., via strong-field ionization^17,18^, photoionization by laser pulses^1,19^ and synchrotron radiation^20–22^, or bombardment with a charged particle (electron^23,24^ or ion^25,26^), while others use pump-probe schemes to obtain time-resolved information on the migration process^1,2,5,12,13,27–30^.

**Figure 1:**
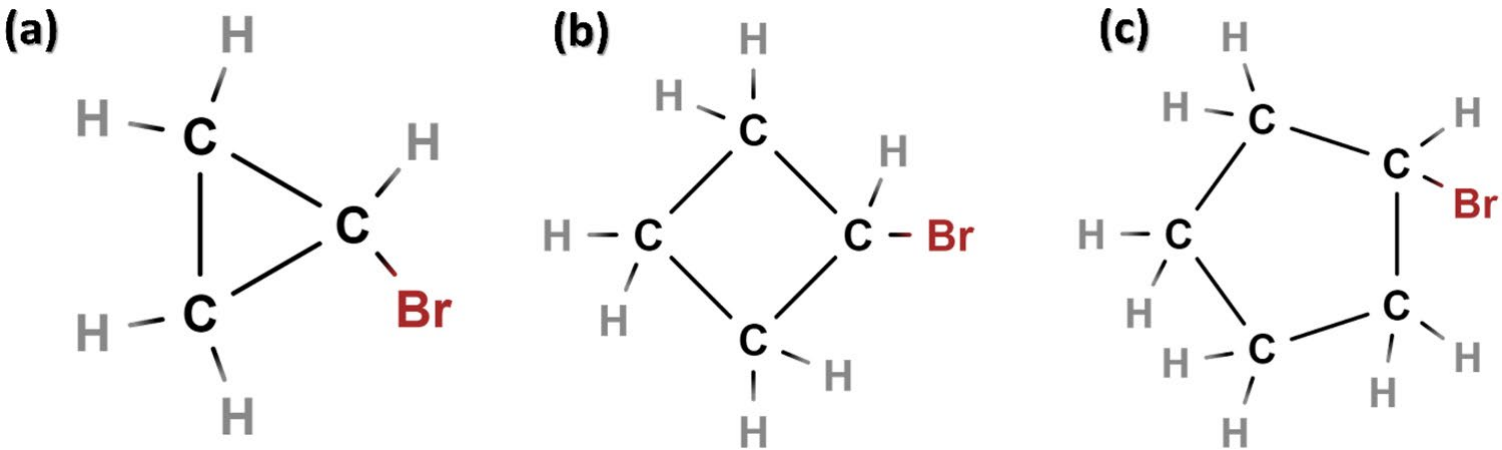
Chemical structure of the molecules discussed in the paper: (**a**) Bromocyclo-propane (BCpro), (**b**) Bromocyclo-butane (BCbut), and (**c**) Bromocyclo-pentane (BCpen). For simplicity, the atomic constituents are drawn in one plane. The full three-dimensional structure is shown in the Supplementary Information.

In this article, we report a systematic study of hydrogen migration processes and fragmentation mechanisms upon inner-shell ionization of the bromine-substituted cyclic hydrocarbons bromocyclo-propane (BCpro, C_3_H_5_Br), bromocyclo-butane (BCbut, C_4_H_7_Br), and bromocyclo–pentane (BCpen, C_5_H_9_Br), which are depicted in Fig. 1. Using a multi-ion coincidence momentum imaging scheme, we investigate fragment ion kinetic energies and momentum correlations and identify a strong correlation between hydrogen migration and sequential fragmentation. Furthermore, we observe that the yield of CH_3_^+^ fragments, whose formation requires hydrogen (or proton) migration, increases with molecular size.

## Results and discussion

**Figure 2:**
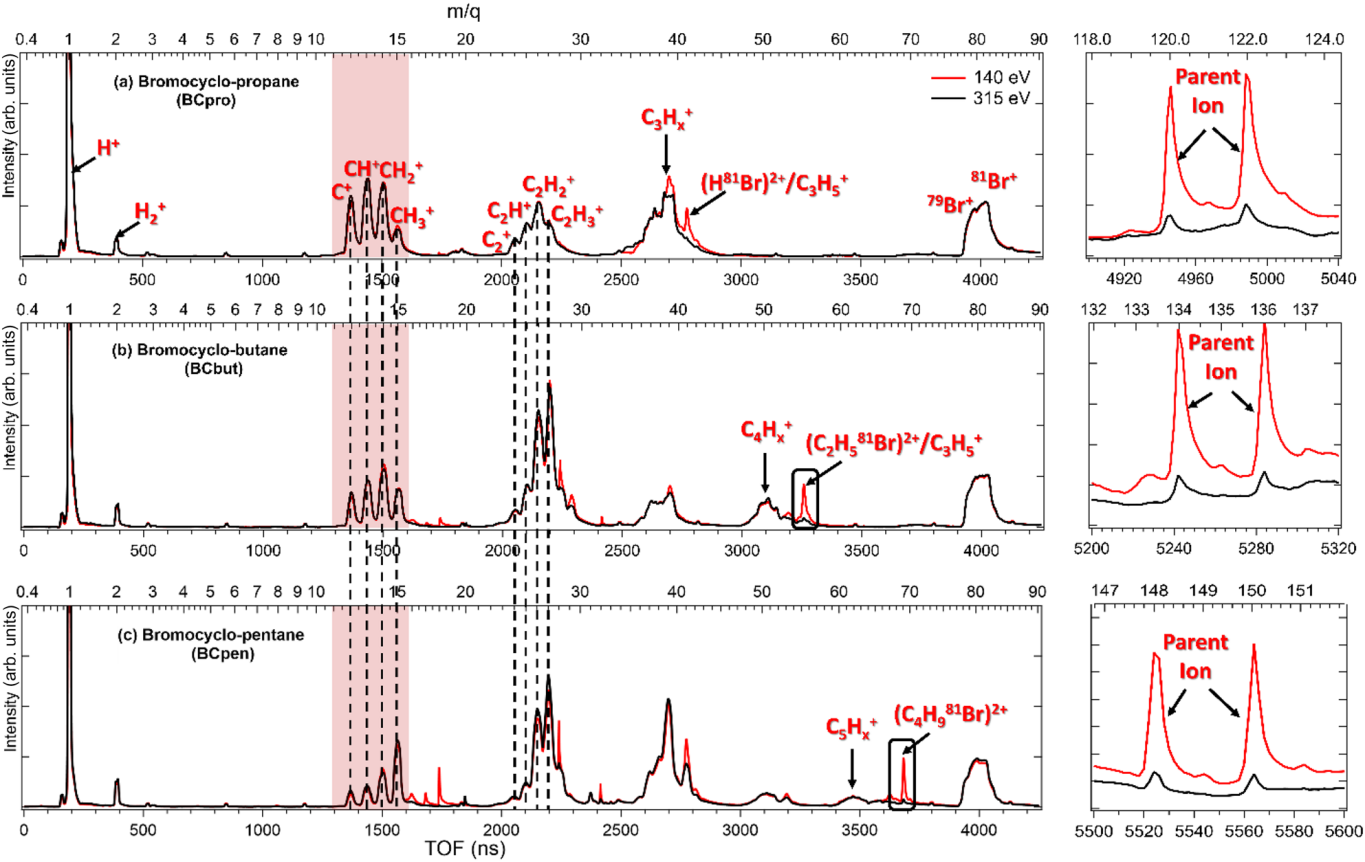
Mass spectra of (**a**) BCpro, (**b**) BCbut, and (**c**) BCpen irradiated at 140 eV (red) and 315 eV (black) photon energy, normalized to have the same integral counts. The mass spectra have no significant peak beyond 90 amu except for two small peaks corresponding to the parent ion, shown in the zoomed-in view on the right.

Figure 2 shows the mass spectra of (a) BCpro, (b) BCbut, and (c) BCpen upon photoionization at two photon energies, 140 eV (approximately 70 eV above the Br(3*d*) ionization threshold) and 315 eV (approximately 25 eV above the C(1*s*) ionization threshold). The spectra have been normalized by their integrated intensity. Inner-shell ionization results in a singly charged ion with a core hole that rapidly relaxes via Auger-Meitner decay^31^, thus leading to a multiply charged molecule that is likely to fragment further into two or more charged and/or neutral fragments. Accordingly, in addition to strong H^+^ and Br^+^ signals (the latter consisting of overlapping peaks of ^79^Br and ^81^Br with approximately equal natural abundance), a sequence of peak groups is observed in the spectra corresponding to singly charged C_n_H_m_^+^ fragments. In BCbut and BCpent, these hydrocarbon fragments with one to three carbons are more abundant than fragments containing four or five carbons. Finally, sharp peaks corresponding to singly charged parent ions and (meta)stable dications HBr^2+^, C_2_H_5_Br^2+^, and C_4_H_9_Br^2+^, the latter highlighted by the black rectangles, are prominently visible at 140 eV but much weaker at 315 eV. These ion species stem primarily from valence (single or double) ionization and are therefore less abundant at the higher photon energy where the ratio of the valence to inner-shell ionization cross sections is smaller^32^. Apart from the relative yield of these parent ions and dications, the mass spectra for each molecule taken at the two different photon energies are similar.

Most of the peaks in the mass spectrum are broadened by the fragments’ kinetic energy resulting from the repulsion (‘Coulomb explosion’) from one or several charged co-fragments, which we will discuss in more detail below. The singly charged parent ion peaks are sharp since these ions have very low kinetic energy. The doubly charged fragments, marked by the black rectangles in Fig. 2, also show relatively narrow peaks, suggesting that they are produced with neutral co-fragment(s) without gaining kinetic energy in the Coulomb explosion. In the plots of the detector hit position as a function of time of flight shown in Fig. S7 in the Supplementary Information, the parent cation and dicationic fragments appear as well localized spots^32^.

**Figure 3:**
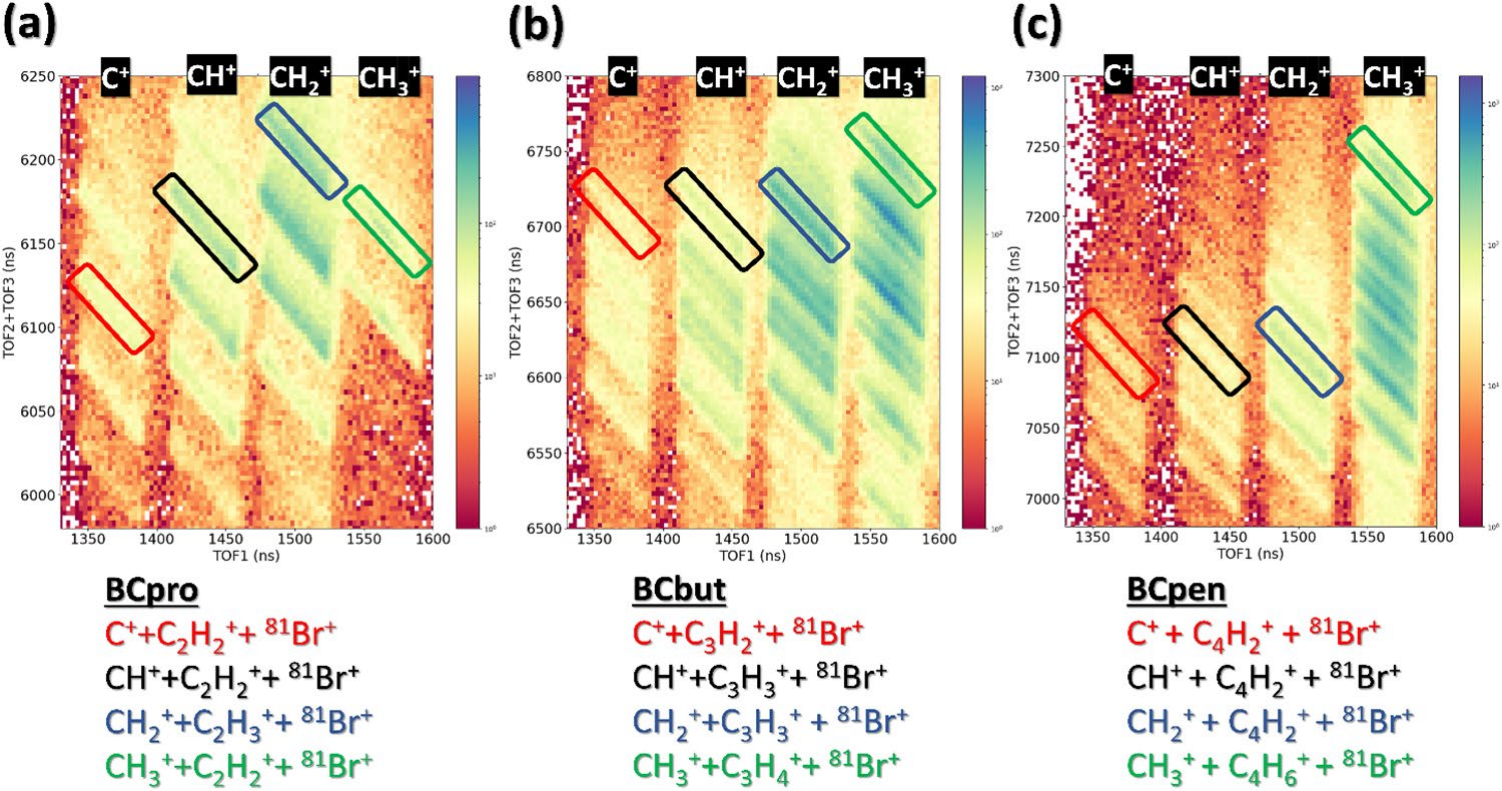
Triple-ion coincidence (TriPICO) plots at 140 eV photon energy for (**a**) BCpro, (**b**) BCbut, and (**c**) BCpen, zoomed-in on the region containing CH_x_^+^ fragments (with x = 0, …, 3) detected in coincidence with a Br^+^ fragment and (b) a C_2_H_x_^+^ (with x = 0, …, 4 ) fragment, (**c**) a C_3_H_x_^+^ (with x = 0, …, 6 ) fragment, and (**d**) a C_4_H_x_^+^ (with x = 0, …, 8) fragment. The fragmentation channels chosen for the further analysis are marked by rectangular regions of interest and identified below each panel.

Comparing the mass spectra for the three different species in more detail, an interesting trend can be seen in the red shaded area highlighted in Fig. 2: The relative abundance of CH_3_^+^ fragments compared to the abundance of C^+^, CH^+^, and CH_2_^+^ fragments increases dramatically as the size of the hydrocarbon ring increases. This is particularly noteworthy since the formation of CH_3_^+^ requires hydrogen migration as there is no carbon atom bound to three hydrogens in the neutral parent molecule (see Fig. 1). Interestingly, this behavior is the opposite of what was observed for linear hydrocarbons^2^, suggesting that it may be related to the strain in the ring molecules, which is highest in BCpro. This trend is also clearly visible in the yield of triple photoion coincidences (TriPICO), i.e., the yield of three ions that were detected in coincidence, shown in Fig. 3, and in the Supplementary Information Figs. S1, S2, and S3. In the following, we will concentrate on data recorded at 140 eV photon energy by further analyzing these TriPICO events. Their kinetic energy distributions and momentum correlations allow further insights into the fragmentation dynamics of the parent trication, which is formed almost exclusively by inner-shell ionization, either by an Auger-Meitner cascade or double-Auger-Meitner process^33,34^, or by direct double photoionization (‘shake-off’)^35^ followed by a single Auger-Meitner decay^31^. The data recorded at 315 eV photon energy shows the same trends as discussed in the following for the 140 eV data.

In the TriPICO plots, the yield of three ions detected in coincidence is plotted as a function of the first ion’s time of flight (TOF1) on the X-axis and the sum of the second and third ions’ time of flight (TOF2 + TOF3) on the Y-axis. Narrow diagonal patterns in TriPICO maps correspond to fragmentation channels in which the sum of the momenta of three charged cations is close to zero. This either corresponds to ‘complete’ channels where the detected ions contain all the atoms in the molecule, or only very little momentum is carried away by a typically neutral and light fragment. This paper mainly concentrates on complete channels as well as those breakups where the bromine and all of the carbon atoms are accounted for in the ions but some of the hydrogens are missing, which may have been emitted as neutral atoms or as protons. The corresponding events are shown in the zoomed-in TriPICO plots in Fig. 3a–c for BCpro, BCbut, and BCpen, respectively. Full TriPICO plots are shown in Figs. S1, S2 and S3 in the Supplementary Information.

**Figure 4:**
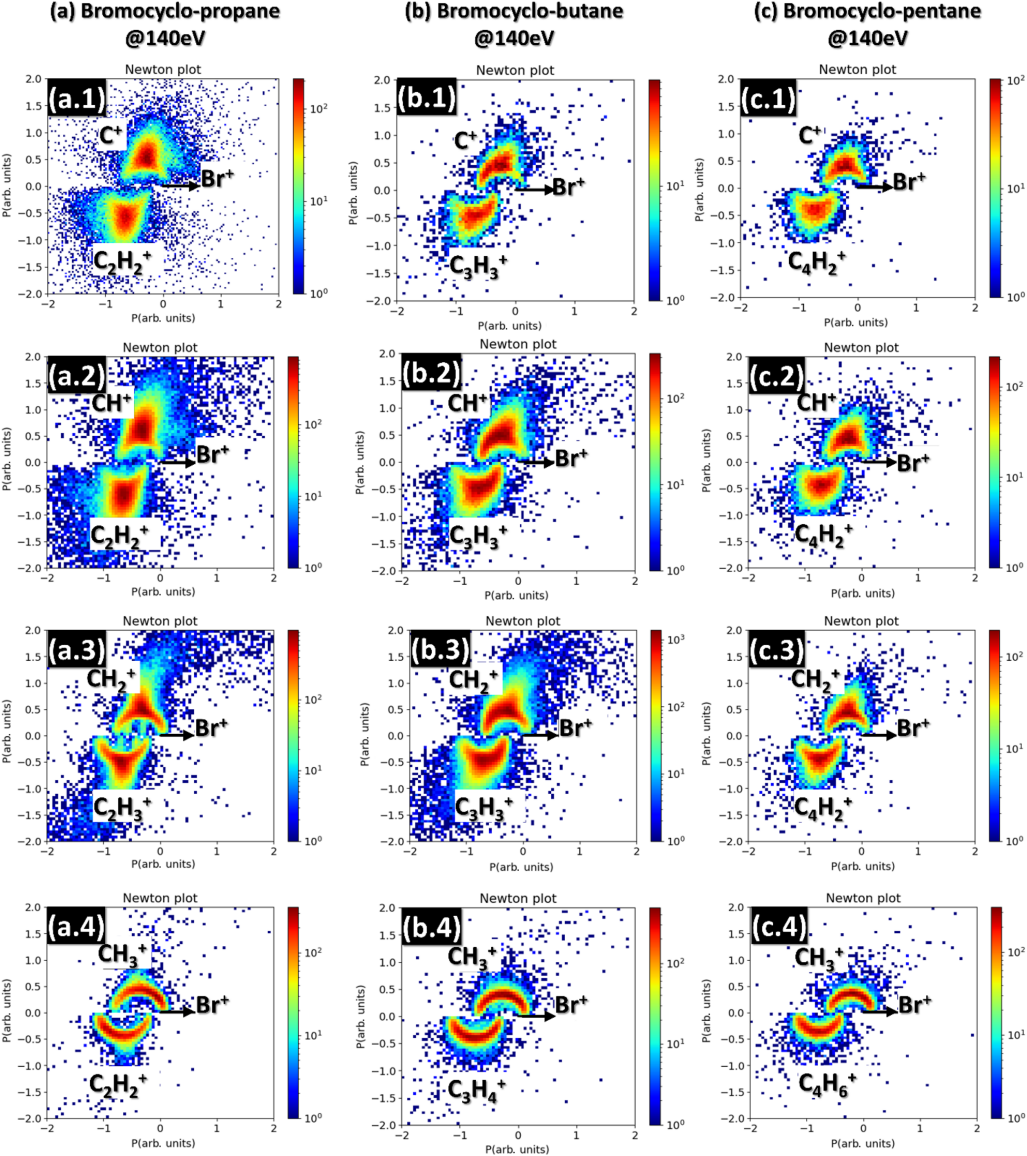
Comparison of Newton plots for the different molecules (all at 140 eV photon energy): (**a**) BCpro (a.1: C^+^ + C_2_H_2_^+^ + ^81^Br^+^), (a.2: CH^+^ + C_2_H_2_^+^ + ^81^Br^+^)_,_ (a.3: CH_2_^+^ + C_2_H_3_^+^ + ^81^Br^+^), (a.4: CH_3_^+^ + C_2_H_2_^+^ + ^81^Br^+^); (**b**) BCbut (b.1: C^+^ + C_3_H_2_^+^ + ^81^Br^+^), (b.2: CH^+^ + C_3_H_3_^+^ + ^81^Br^+^), (b.3: CH_2_^+^ + C_3_H_3_^+^ + ^81^Br^+^), (a.4: CH_3_^+^ + C_3_H_4_^+^ + ^81^Br^+^); (**c**) BCpen (c.1: C^+^ + C_4_H_2_^+^ + ^81^Br^+^), (c.2: CH^+^ + C_4_H_2_^+^ + ^81^Br^+^), (c.3: CH_2_^+^ + C_4_H_2_^+^ + ^81^Br^+^), (c.4: CH_3_^+^ + C_4_H_6_^+^ + ^81^Br^+^). In each plot, all fragment momenta are normalized such that the magnitude of the Br^+^ fragment momentum is unity.

In order to gain further information about the fragmentation mechanism, TriPICO data is often represented as a Newton plot, which visualizes the momentum correlation of the three charged fragments. It is constructed by plotting the momentum (both magnitude and angle) of two of the fragments with respect to a third fragment that is chosen as a reference to define the x-axis in the plot and whose momentum is set to unity. Figure 4 shows the Newton plots for several of the channels of interest with the Br^+^ fragment as a reference, and the momentum of the first fragment plotted in the upper half (y > 0) of the plot and the momentum of the second fragment on the lower half (y < 0). The columns show the plots for different molecules (from left to right BCpro, BCbut, and BCpen) and rows from top to bottom show channels where the lightest ion is C^+^, CH^+^, CH_2_^+^, and CH_3_^+^, respectively.

As shown in previous work^1,2,5,36–40^, Newton plots often have characteristic features that are the result of concerted and sequential fragmentation processes. Sequential fragmentation, where the bond breaking that leads to the formation of the three fragments occurs in two distinct steps separated in time by more than the rotational period of the intermediate fragment, leads to a semi-circular structure, as seen in the bottom row of Fig. 4. On the other hand, concerted fragmentation, where the bond breaking occurs simultaneously or with a delay less than the rotational period of the intermediate, leads to more localized maxima, which are predominant in the top row of Fig. 4. Based on the inspection of the Newton plots, we can thus conclude that the fragmentation process leading to the formation of a CH_3_^+^ fragment is purely sequential in all three molecules considered here, while both sequential and concerted processes are contributing to the formation of C^+^, CH^+^, and CH_2_^+^ fragments, with the relative contribution of sequential processes apparently increasing for the latter. Furthermore, we can also conclude that the sequential processes almost exclusively involve the emission of the Br^+^ fragment in the first step and a breakup of the remaining C_n_H_x_^2+^ dication in the second step, e.g., C_3_H_5_Br^3+^ → C_3_H_5_^2+^ + ^81^Br^+^ → CH_3_^+^ + C_2_H_2_^+^ + ^81^Br^+^, since a different sequence would lead to distinctly different signatures in the Newton plots^40^. The identification of sequential and concerted fragmentation dynamics is often also done on the basis of Dalitz plots^1^, which are shown in Fig. S4 in the Supplementary Information, and from which similar conclusions can be drawn.

**Figure 5:**
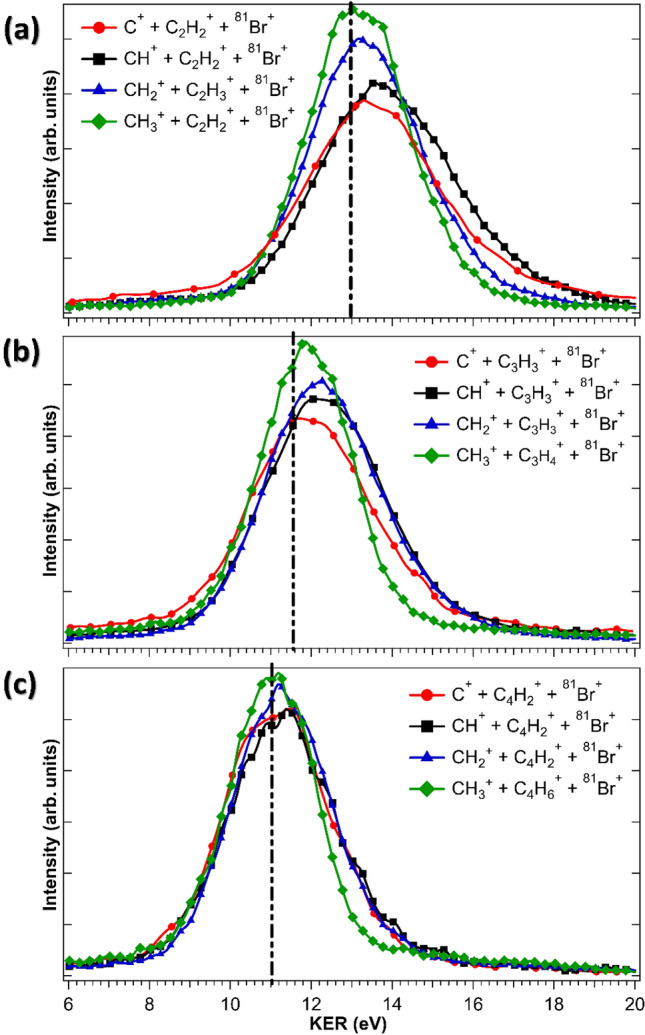
Comparison of the KER distributions for different fragmentation channels in (**a**) BCpro, (**b**) BCbut, and (**c**) BCpen. All distributions are normalized to have equal integral yield. The vertical black dash-dotted lines show the values obtained from Coulomb explosion simulations (see text).

**Figure 6:**
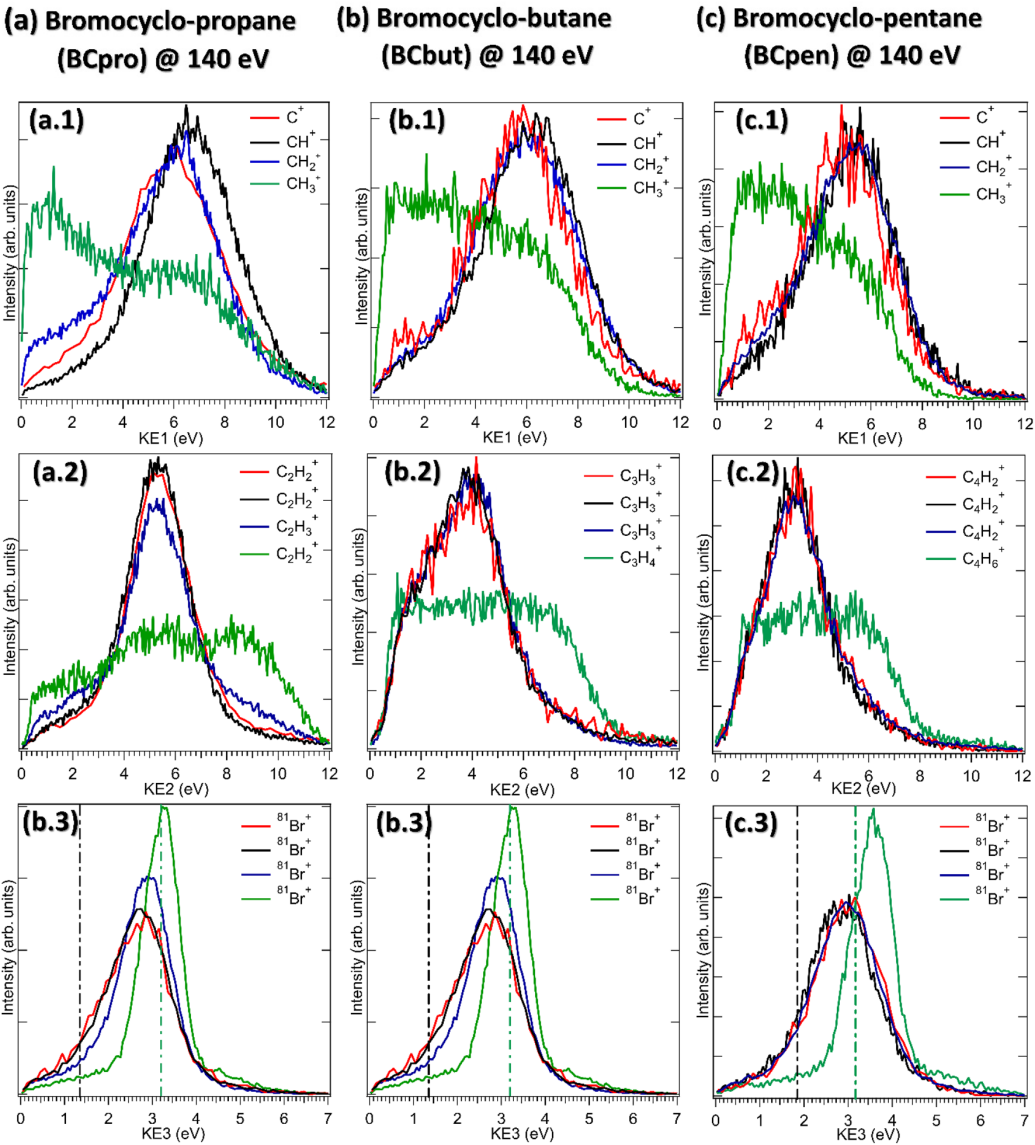
The kinetic energies of the individual fragments in different fragmentation channels of (**a**) BCpro, (**b**) BCbut, and (**c**) BCpen. The same color in top, middle and bottom row marks the fragments belonging to the same fragmentation channel. All distributions are normalized to have equal integral yield. The vertical, dash-dotted lines in the bottom row show the simulated Br^+^ KE values for concerted (black) and sequential (green) fragmentation.

Further information about the structure of the multiply charged ions and the reaction energetics and dynamics of the unimolecular fragmentation can also be obtained from the kinetic energy release (KER) distributions^41^, which are shown in Fig. 5, and the fragment kinetic energy distributions shown in Fig. 6. Figure 5 compares the KER distributions for all complete fragmentation channels containing CH_*x*_^+^ fragments (with *x* = 0, …, 3) in the three different bromocyclo compounds. While the center of the distributions appears to be rather independent of *x*, a clear trend is seen when comparing the KER for the three different molecules, with the largest KER in the smallest molecule, BCpro, and the smallest KER in the largest molecule, BCpen. This can be intuitively rationalized when assuming that the KER can be approximated as the electrostatic potential energy of three point-charges distributed on a nearly ring-shaped molecule, which would lead to a smaller energy the larger the diameter of the ring. To link this simple picture more directly and quantitatively to the actual molecular structure, we performed classical Coulomb explosion simulations for concerted fragmentation starting from the equilibrium geometry of each molecule and assuming that one point charge is located on the Br atom and the other two point charges are placed on two of the other atoms in the molecule such that the total electrostatic potential energy is minimized (see Methods and Supplementary Information for further details on the Coulomb explosion simulations and the assumed position of the point charges). The resulting simulated KERs indicated with a dash-dotted line in Fig. 4 clearly reproduce the trend of a smaller KER the larger the molecule and lie close to the center values of each experimental distribution, thus corroborating the intuitive picture.

Comparing the KER distributions for different channels within the same molecule, it is evident that the fragmentation channels producing CH_3_^+^ fragments consistently have narrower distributions than those producing C^+^, CH^+^, and CH_2_^+^ fragments. Combining this with the observations from Fig. 4 suggests that this is because the channels producing CH_3_^+^ are formed via a single mechanism (namely sequential fragmentation), while the other channels contain a mix of sequential and concerted fragmentation, which broadens the KER distributions. However, we cannot exclude that some of the differences also stem from the fact that many of the channels with C^+^, CH^+^, and CH_2_^+^ are missing one or more hydrogens, since we selected the most abundant TriPICO channels for this comparison.

To further investigate the fragmentation dynamics, we compare the individual fragment kinetic energy (KE) distributions in Fig. 6. At first sight, several clear trends are visible: For the CH_3_^+^ containing fragmentation channels (shown in green), the KE distributions of CH_3_^+^ and the other hydrocarbon fragment are much broader than for the other fragmentation channels. In contrast, the corresponding Br^+^ fragment, shown in the bottom row, has a narrow kinetic energy distribution with higher central energy than the other channels, which also shifts towards higher energies the larger the molecule.

All these observations can, again, be explained with the sequential mechanism leading to this channel. The initial ejection of the Br^+^ fragment in a sequential breakup leads to its higher KE as compared to the case of concerted breakup since it is repelled off a single, massive, doubly charged fragment. Furthermore, due to momentum conservation, the fraction of the total kinetic energy imparted onto the Br^+^ fragment increases for a more massive co-fragment, i.e., the larger the molecule. To quantitatively confirm this picture and, in particular, the trends observed in the Br^+^ KE, the KE value obtained from the Coulomb explosion simulations for sequential and concerted fragmentation for the same charge distributions as described above are shown as vertical dash-dotted lines. The simulations agree well with the overall trends but consistently underestimate the observed Br^+^ KEs, confirming our assignment of concerted and sequential channels but also exposing the expected limitations of the simple model for the initial charge distribution, with point charges at the “far ends” of the molecule in its equilibrium geometry^42,43^.

For the case of sequential fragmentation, the momenta of the two hydrocarbon fragments are the sum of the momenta imparted in the first step and the second step. Since the intermediate fragment rotates before it fragments further, the momenta from both individual steps can be parallel (leading to large final momentum and, thus, large fragment KE), antiparallel (leading to small final momentum and KE), or any angle in between (leading to an intermediate KE), which therefore leads to a very broad KE distribution.

## Conclusions

We have shown that Br(3*d*) and C(1*s*) inner-shell ionization of the brominated cyclic hydrocarbons bromocyclo-propane, bromocyclo-butane, and bromocyclo-pentane leads to a substantial amount of CH_3_^+^ fragments, which are produced via intramolecular hydrogen (or proton) migration. The relative abundance of the hydrogen (or proton) migration increases with molecular size and, thus, decreasing ring strain. Furthermore, by analyzing fragment ion momentum correlations of three-body fragmentation channels of the trication, we have found that CH_3_^+^ fragments in those channels are almost exclusively formed by a sequential fragmentation pathway that proceeds via initial C–Br bond cleavage and formation of a long-lived propane, butane, or pentane dication. While our experimental observations and analysis cannot determine the geometry of the initial trication nor the intermediate dications (i.e., whether they remain cyclic or undergo ring opening), we observe a clear dependence of the experimentally observed kinetic energy releases and fragment kinetic energies on the molecular size, which are rationalized with the help of classical Coulomb explosion simulations. Further information about the time scale of the hydrogen (or proton) migration and the geometry of the intermediate dications may be obtained from future time-resolved experiments that could employ ion mass spectrometry or ion imaging with disruptive probing^44,45^, Coulomb explosion imaging^46^, or ultrafast electron^47^ or X-ray^48^ scattering.

## Methods:

### Experimental details

The sample molecules, purchased from Sigma Aldrich, have a purity of ≥ 98.5% for BCpro (C_3_H_5_Br), ≥ 95.5% for BCbut (C_4_H_7_Br), and ≥ 98.0% for BCpen (C_5_H_9_Br), according to the vendor's gas chromatography analysis. The samples are liquid at room temperature and were brought into the gas phase via supersonic expansion through a 30-µm aperture without further heating as their room temperature vapor pressures (148.2 ± 0.1 mmHg for BCpro, 32.0 ± 0.2 mmHg for BCbut, and 9.7 ± 0.2 mmHg for BCpen, according to the predictions for 25 °C published in the ChemSpider molecules database^49^) were sufficient to form a molecular beam without the use of a carrier gas. After the expansion, the molecular beam passes through a 500-µm skimmer and crosses a beam of monochromatic, linearly polarized synchrotron radiation. The experiment was conducted at beamline 8.0.1.2 of the Advanced Source Light (ALS) at Lawrence Berkeley National Laboratory while the storage ring was running in the 2-bunch top-off operation mode with a bunch spacing of 328 ns. Electrons and ions produced by the interaction of the synchrotron radiation with the molecular beam were detected in coincidence using a double-sided velocity map imaging (VMI) spectrometer, which is described in detail in prior publications^36,37,50^. A schematic of the setup is shown in Fig. S5 in the Supplementary Information. Our double-sided coincidence VMI is different from a conventional VMI setup^51^ in the sense that it can detect both electrons and ions simultaneously, and that it is using microchannel plate (MCP) detectors equipped with multi-hit delay line anodes (RoentDek DLD80 for electrons and HEX80 for ions) rather than phosphor screens to record the time and impact position of the charged fragments. The signals from the MCPs and delay lines were recorded using multi-hit time-to-digital (TDCs) converters with a resolution of < 100 ps and a multi-hit dead-time of < 10 ps that were triggered by the detection of the first electron (which could be either a photoelectron or Auger-Meitner electron), which reaches the detector after a flight time of approximately 5 ns and which serves as a start for the time-of-flight measurement of the ions.

As the electric field applied to the spectrometer is not homogenous, the SIMION software package^52^ was used to calculate the three-dimensional momentum vectors of the detected ions from their time of flight and hit positions. The procedure is described in detail by Ablikim et al.^36,50^, and only a short description is given here. First, P_x_ and P_y_, the momentum vector components parallel to the detector, and P_z_, the momentum vector component perpendicular to the detector, were constructed from each ion's hit position and time of flight spread, respectively, using conversion factors determined from the SIMION simulation. For the coincidence analysis, only those events where the component-wise sum of all fragment ion momenta was close to zero (with full width at half maximum of ± 20 a.u.) were selected in order to suppress false coincidence events. The three-dimensional momentum vectors were then used to calculate the kinetic energy releases and momentum correlations^36,50^.

With the VMI lens voltages used for this measurement, it was possible to detect singly charged ions with kinetic energies up to 15 eV over the full solid angle.

### Coulomb explosion simulation

The total Coulomb potential energy E_tot_ (in units of eV) of a multiply charged molecule due to a distribution of N point charges can be expressed as:

1 $$E_{tot} \left( {{\text{eV}}} \right) = 27.21\mathop \sum \limits_{i \ne j}^{N} \frac{{q_{i} q_{j} }}{{\left| {r_{i} - r_{j} } \right|}},$$

where the charges *q*_*i*_ and *q*_*j*_ (in atomic units, a.u.) are separated by distance |*r*_*i*_ − *r*_*j*_| (in a.u.)^36,38^. We evaluate the positions and momenta of the fragments at any given time during the three-body fragmentation by numerically solving the classical equations of motion under the influence of the Coulomb field and using several simplifying assumptions. Those assumptions are: (i) the Coulomb explosion of a molecule is governed by a purely Coulombic repulsion between point charges, (ii) the explosion starts from the equilibrium geometry of the neutral molecule, and (iii) there is no energy stored in the internal degrees of freedom of the fragments or the transient molecular ion^42^. These Coulomb explosion simulations (CES) are performed on the ground state geometries of parent BCpro, BCbut, and BCpen molecules optimized at the* ω*B97X-D/aug-cc-pVDZ level of theory using the Gaussian 16 suite of programs^53^ without any constraints. The optimized geometries are shown in Supplementary Information Fig. S6 and the corresponding optimized cartesian coordinates in Table S1, S2, and S3.

We follow the commonly used terminology to classify the different pathways leading to three-body fragmentation^54^: If the delay (Δτ) between two bond breaking processes is less than the mean rotational period, τ_rot_, of the intermediate fragment, i.e., Δτ/τ_rot_ < 1, the process is called a concerted breakup. The limiting case when both bonds break simultaneously, i.e., Δτ/τ_rot_ = 0, is called synchronous concerted, while an asynchronous concerted breakup is defined as 0 < Δτ/τ_rot_ < 1. If Δτ/τ_rot_ > 1, the bond breaking is called sequential. Here, we perform the CES for synchronous concerted and sequential bond-breaking processes. In order to minimize the initial Coulomb potential, in both cases, three point charges of + *e* each are placed on the three atoms in the molecule that will yield the longest distances between the charges, as shown in Table S4. The results for an alternative placement of the point charges restricted only to the Br and C atoms is shown in Table S5. The positions and momenta at any instant after the fragmentation are calculated by numerically solving the classical equations of motion of point charges in a Coulomb field using a 4th order Runge–Kutta method. For simplicity, we simulate only the first step of the sequential breakup, i.e., the C–Br bond breakup, in order to compare the kinetic energies of Br^+^ ions in the sequential breakup process with those of the concerted one.

## Data availibility

The datasets used and/or analysed during the current study available from the corresponding author on reasonable request.

## Supplementary Information

Supplementary Information.
