# Supplementary material for: Hydrogen migration in inner-shell ionized halogenated cyclic hydrocarbons
Source: Sci Rep. 2023 Feb 6;13:2107. doi: 10.1038/s41598-023-28694-x (PMC9902455; doi:10.1038/s41598-023-28694-x)
Supplement: Supplementary file 1 — Supplementary Information. [file 41598_2023_28694_MOESM1_ESM.pdf]

# Supplementary Information

## Hydrogen migration in inner-shell ionized halogenated cyclic hydrocarbons

Abdul Rahman Abid[1,2,3], Surjendu Bhattacharyya[1], Anbu Selvam Venkatachalam[1], Shashank Pathak[1], Keyu Chen[1], Huynh Van Sa Lam[1], Kurtis Borne[1], Debadarshini Mishra[4], René C. Bilodeau[4], Ileana Dumitriu[5], Nora Berrah[4], Minna Patanen[2], Daniel Rolles[1]

*[1]J. R. Macdonald Laboratory, Department of Physics, Kansas State University, Manhattan, KS 66506, USA*
*[2]Nano and Molecular Systems Research Unit, University of Oulu, Finland*
*[3]Department of Physics and Astronomy, Aarhus University, 8000 Aarhus, Denmark*
*[4]Department of Physics, University of Connecticut, Storrs, CT 06269, USA*
*[5]Hobart and William Smith Colleges, Geneva, New York 14456, USA*

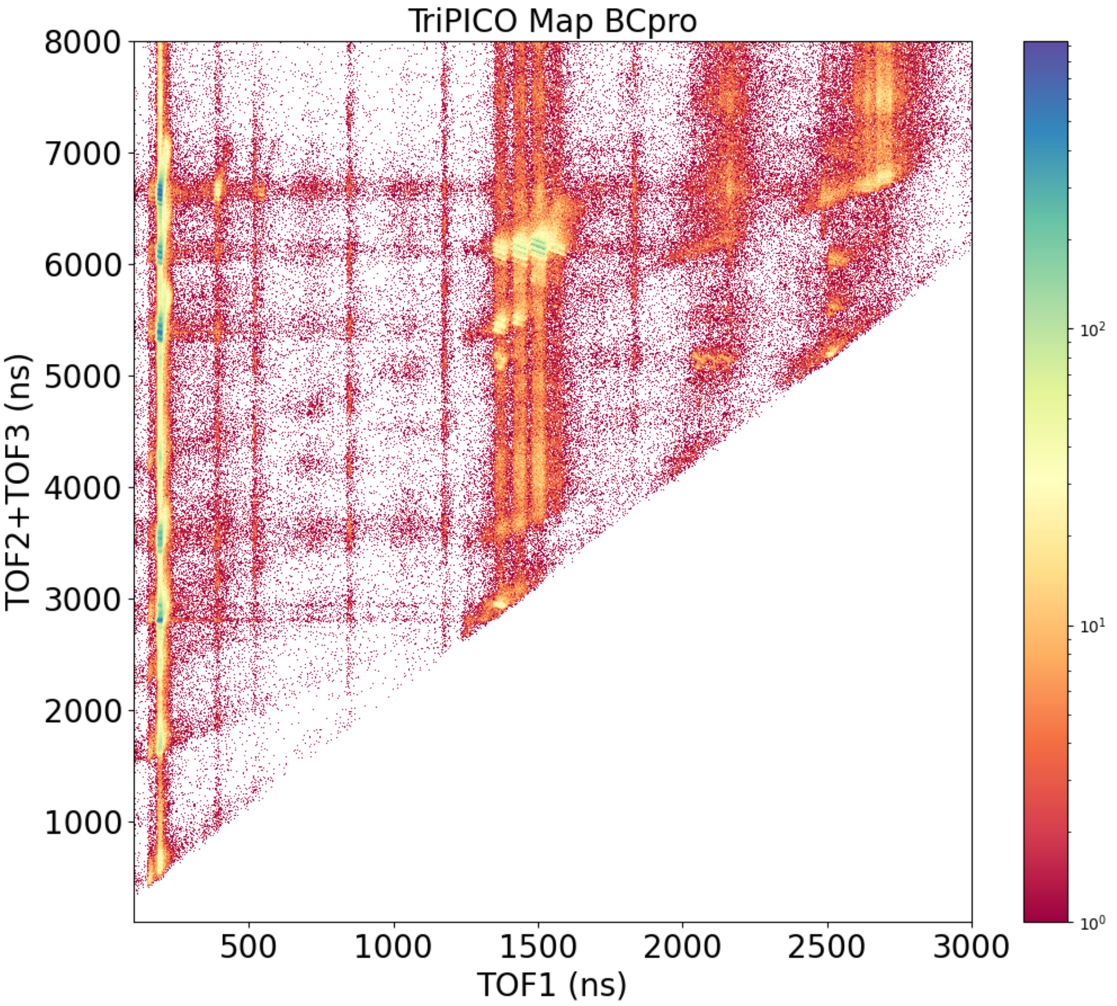


**Figure S1:** Triple-ion coincidence (TriPICO) map for BCpro. The X-axis is the time-of-flight of the first ion (TOF1), and Y-axis is the time-of-flight of the second plus third ion (TOF2+TOF3). The color indicates the coincidence ion yield in counts.

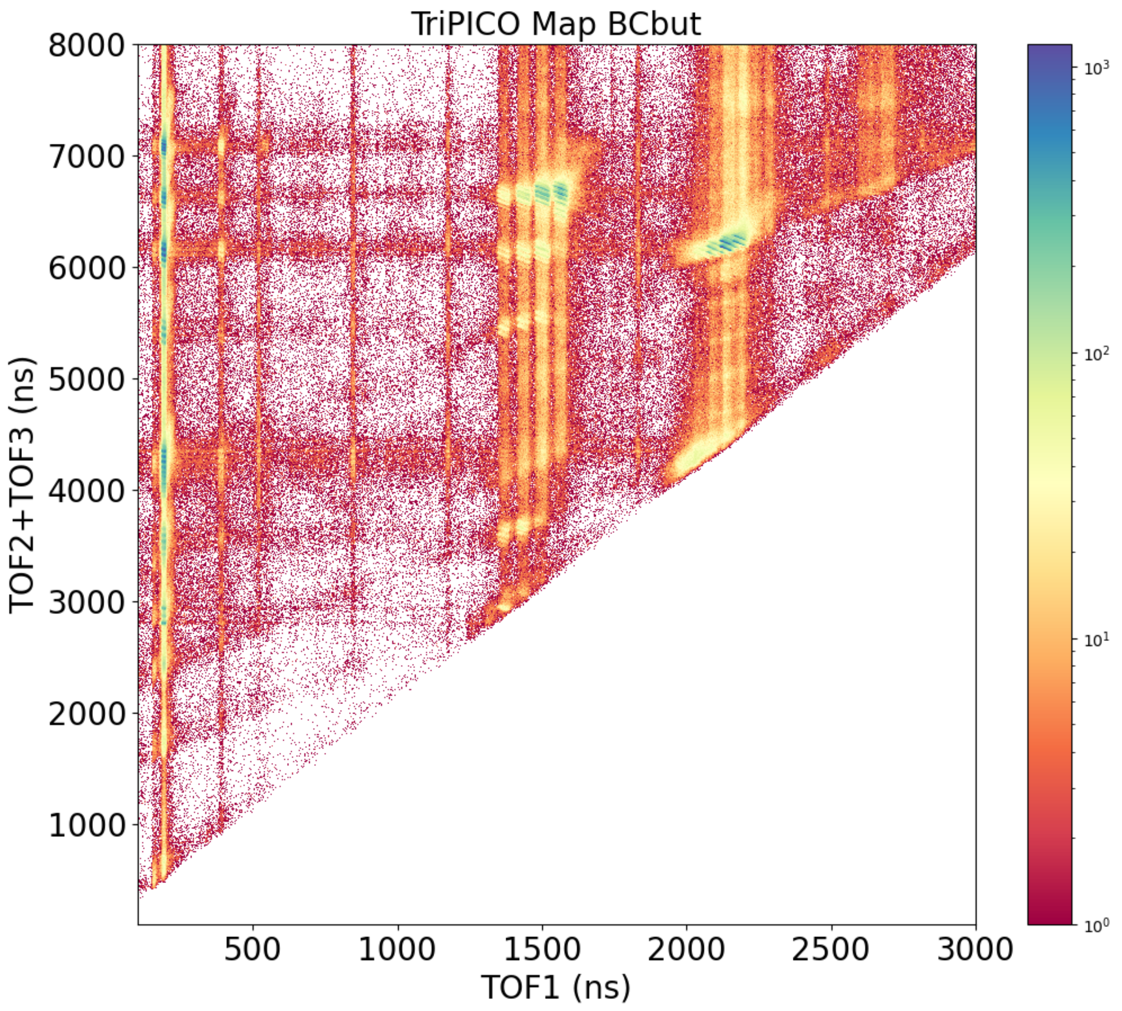


**Figure S2:** Triple-ion coincidence (TriPICO) map for BCbut. The X-axis is the time-of-flight of the first ion (TOF1), and Y-axis is the time-of-flight of the second plus third ion (TOF2+TOF3). The color indicates the coincidence ion yield in counts.

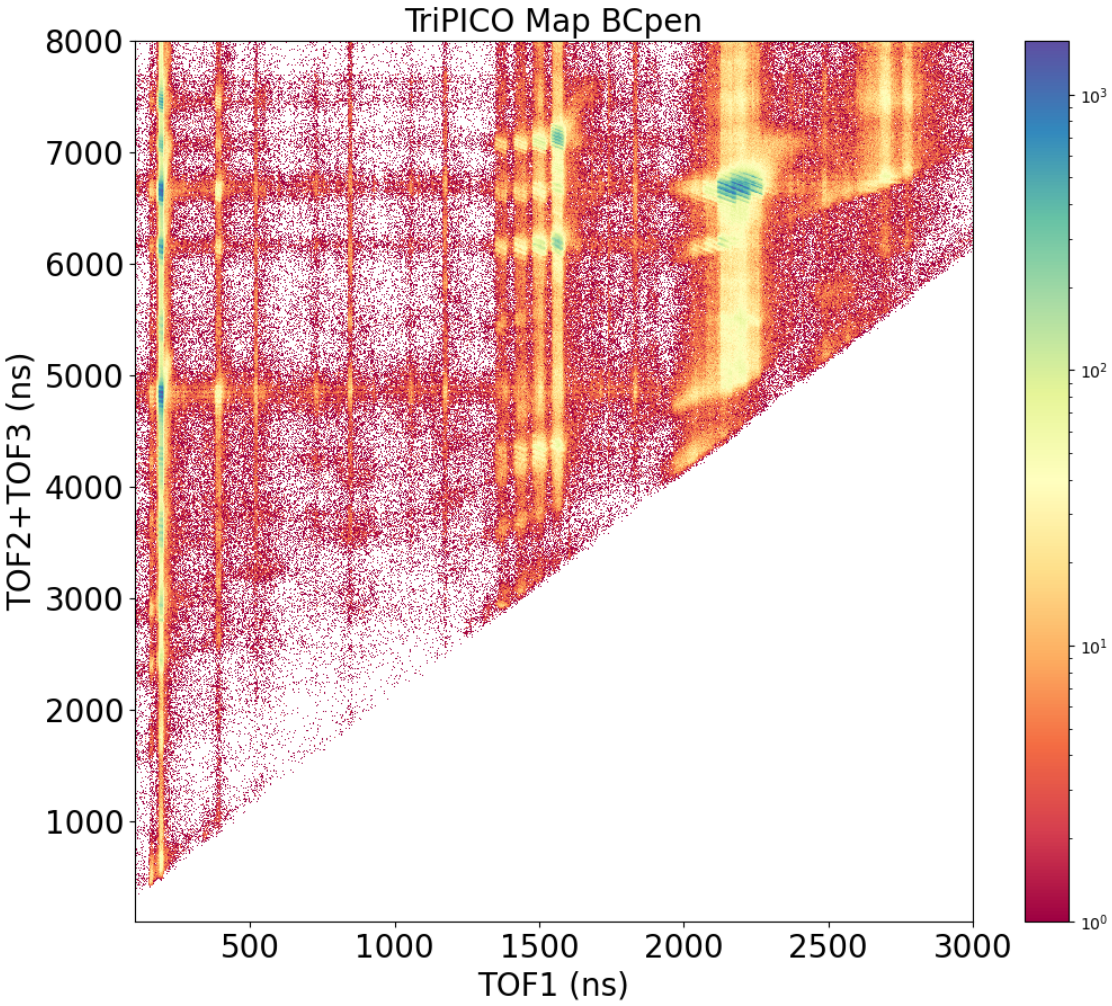


**Figure S3:** Triple-ion coincidence (TriPICO) map for BCpen. The X-axis is the time-of-flight of the first ion (TOF1), and Y-axis is the time-of-flight of the second plus third ion (TOF2+TOF3). The color indicates the coincidence ion yield in counts.

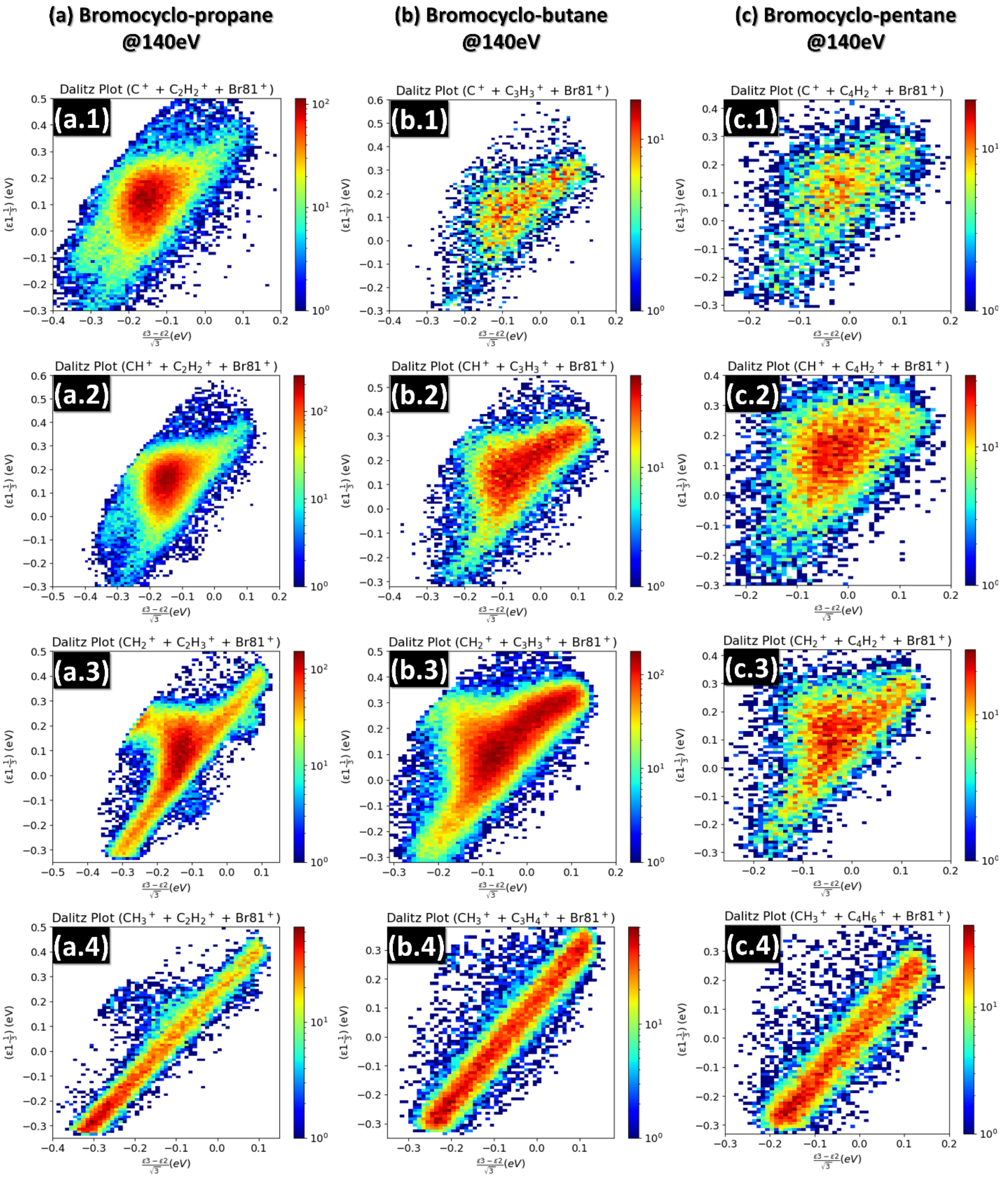


**Figure S4:** Dalitz plots (at 140 eV photon energy) for (a) BCpro (a.1: $C^+ + C_2H_2^+ + {}^{81}Br^+$), (a.2: $CH^+ + C_2H_2^+ + {}^{81}Br^+$), (a.3: $CH_2^+ + C_2H_3^+ + {}^{81}Br^+$), (a.4: $CH_3^+ + C_2H_2^+ + {}^{81}Br^+$); (b) BCbut (b.1: $C^+ + C_3H_2^+ + {}^{81}Br^+$), (b.2: $CH^+ + C_3H_3^+ + {}^{81}Br^+$), (b.3: $CH_2^+ + C_3H_3^+ + {}^{81}Br^+$), (b.4: $CH_3^+ + C_3H_4^+ + {}^{81}Br^+$); (c) BCpen (c.1: $C^+ + C_4H_2^+ + {}^{81}Br^+$), (c.2: $CH^+ + C_4H_2^+ + {}^{81}Br^+$), (c.3: $CH_2^+ + C_4H_2^+ + {}^{81}Br^+$), (c.4: $CH_3^+ + C_4H_6^+ + {}^{81}Br^+$). A Dalitz plot[1] visualizes the energy correlation between the different fragments generated during the fragmentation of the parent molecule. It is generated by plotting the difference of the kinetic energies of the second and third

fragments along the X-axis ($\frac{\varepsilon_3-\varepsilon_2}{\sqrt{3}}$), and the kinetic energy of the first fragment ($\varepsilon_1 - \frac{1}{3}$) along Y-axis. Here, $\varepsilon_i$ is the kinetic energy of an individual fragment divided by the total kinetic energy released during fragmentation ($\varepsilon_i = \frac{KE_i}{KER}$).[2] Energy conservation requires that all events lie in a specific circular region. In a Dalitz plot, the phase space density is constant compared to the final-state phase space density. Therefore, all structures that appear in the Dalitz plots are purely from the dynamics of the process.[3] A band-like structure is a signature of sequential decay because of the large energy difference between the two fragments. A spot-like structure is a signature of concerted decay because of the small energy difference between the two fragments. The Dalitz plots shown above indicate the presence of both concerted and sequential decays and suggest that the fragmentation process leading to the formation of a $CH_3^+$ fragment is purely sequential, while both sequential and concerted processes contribute to the formation of $C^+$, $CH^+$, and $CH_2^+$ fragments.

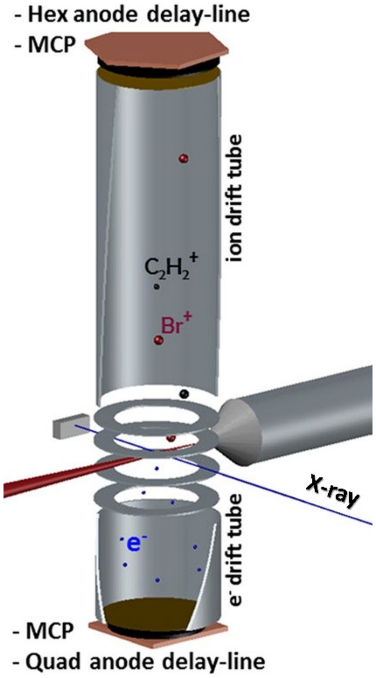


**Figure S5:** Schematics of the double-sided coincidence velocity map imaging (VMI) spectrometer and the positions of the molecular beam and X-ray beam.[4]

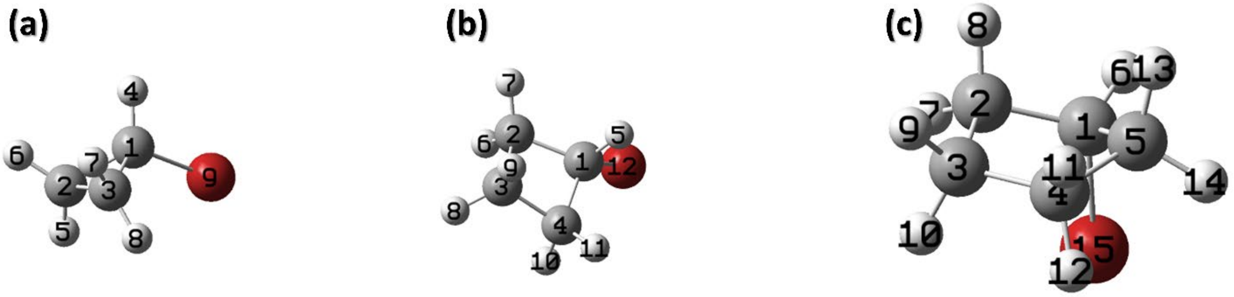


**Figure S6:** Optimized geometries of (a)BCpro, (b)BCbut, and (c)BCpen used to perform the Coulomb explosion simulation. The different colors indicate different atoms: red=bromine, dark grey=carbon, light gray=hydrogen.

**(a) BCpro TOF Vs. Y hit position**

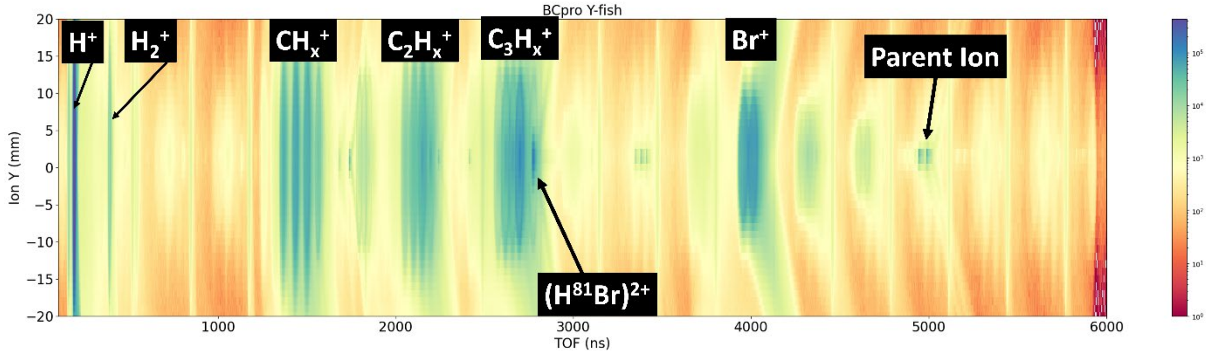


**(b) BCbut TOF Vs. Y hit position**

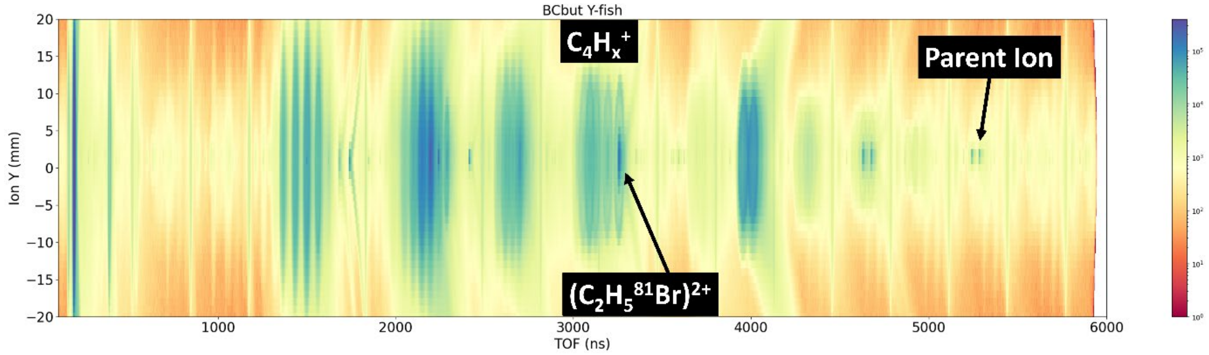


**(c) BCpen TOF Vs. Y hit position**

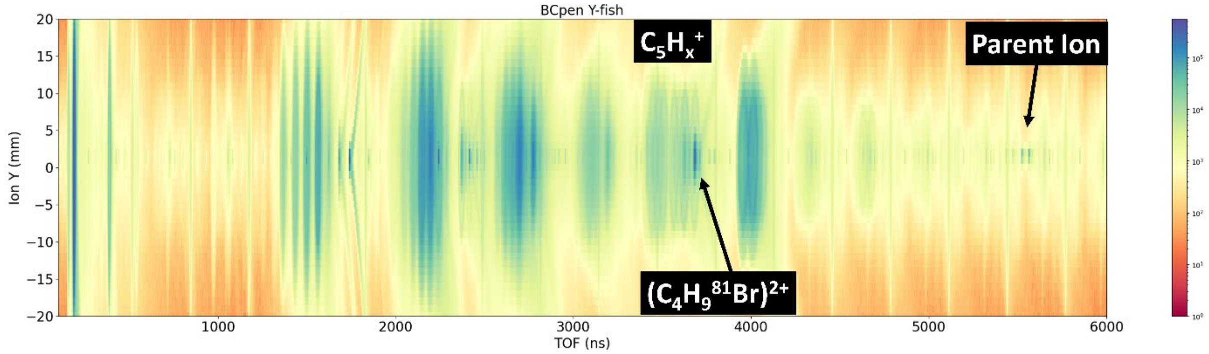


**Figure S7:** Plots of the ion yield as a function of Time-of-Flight (TOF) and detector hit position Y for (a) BCpro, (b) BCbut, and (c) BCpen.

**Table S1:** Cartesian coordinates (in Ångström) for optimized bromocyclo-propane at $\omega$B97X-D/aug-cc-pVDZ level.

| | | | |
|---|---|---|---|
| C | 0.796717 | 0.000000 | 0.575668 |
| C | 1.837216 | -0.757742 | -0.190181 |
| C | 1.837216 | 0.757742 | -0.190181 |
| H | 0.828986 | 0.000000 | 1.662211 |
| H | 1.525736 | -1.256264 | -1.105925 |
| H | 2.596143 | -1.284762 | 0.386479 |
| H | 2.596143 | 1.284762 | 0.386479 |
| H | 1.525736 | 1.256264 | -1.105925 |
| Br | -1.025704 | 0.000000 | -0.039862 |

**Table S2:** Cartesian coordinates (in Ångström) for optimized bromocyclo-butane at $\omega$B97X-D/aug-cc-pVDZ level.

| | | | |
|---|---|---|---|
| C | 0.508587 | 0.000000 | 0.497615 |
| C | 1.434989 | -1.084120 | -0.073548 |
| C | 2.544943 | 0.000000 | -0.122637 |
| C | 1.434989 | 1.084120 | -0.073548 |
| H | 0.489944 | 0.000000 | 1.591660 |
| H | 1.123830 | -1.380664 | -1.082066 |
| H | 1.595234 | -1.978417 | 0.538436 |
| H | 3.202608 | 0.000000 | -0.998661 |
| H | 3.157895 | 0.000000 | 0.786908 |
| H | 1.123830 | 1.380664 | -1.082066 |
| H | 1.595234 | 1.978417 | 0.538436 |
| Br | -1.366561 | 0.000000 | -0.047427 |

**Table S3:** Cartesian coordinates (in Ångström) for optimized bromocyclo-pentane at $\omega$B97X-D/aug-cc-pVDZ level.

| | | | |
|---|---|---|---|
| C | -0.216721 | 0.000131 | 0.941726 |
| C | -1.096132 | 1.197861 | 0.598385 |
| C | -1.861015 | 0.776384 | -0.672062 |
| C | -1.860700 | -0.776666 | -0.672017 |
| C | -1.096097 | -1.197710 | 0.598709 |
| H | 0.162511 | 0.000280 | 1.966813 |
| H | -0.534173 | 2.131171 | 0.486870 |
| H | -1.796409 | 1.330202 | 1.438261 |
| H | -2.875890 | 1.191472 | -0.678597 |
| H | -1.350951 | 1.158479 | -1.564303 |
| H | -2.875384 | -1.192209 | -0.678968 |
| H | -1.350074 | -1.158577 | -1.564018 |
| H | -1.796542 | -1.329666 | 1.438511 |
| H | -0.534162 | -2.131092 | 0.487662 |
| Br | 1.421002 | -0.000002 | -0.174305 |

**Table S4:** Coulomb explosion simulations for concerted and sequential breakup assuming a charge distribution that minimizes the initial Coulomb potential energy.

<table>
<tr><td colspan="4">Bromocyclo-propane (BCpro)</td></tr>
<tr><td rowspan="7">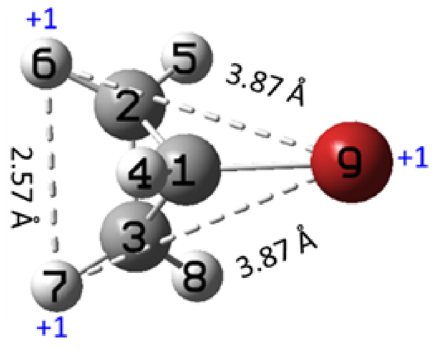
</td><td colspan="3">$CH_2^+ + C_2H_3^+ + {}^{81}Br^+$</td></tr>
<tr><td></td><td>concerted</td><td>Sequential</td></tr>
<tr><td>Bonds break</td><td>$C_{(1)}$-$Br_{(9)}$<br>$C_{(1)}$-$C_{(2)}$<br>$C_{(2)}$-$C_{(3)}$</td><td>Step-1:<br>$C_{(1)}$-$Br_{(9)}$</td></tr>
<tr><td>KE($CH_2^+$)/eV</td><td>7.06</td><td></td></tr>
<tr><td>KE($C_2H_3^+$)/eV</td><td>4.80</td><td></td></tr>
<tr><td>KE($^{81}Br^+$)/eV</td><td>1.20</td><td>2.51</td></tr>
<tr><td>KER/eV</td><td>13.06</td><td></td></tr>
<tr><td colspan="4">Bromocyclo-butane (BCbut)</td></tr>
<tr><td rowspan="6">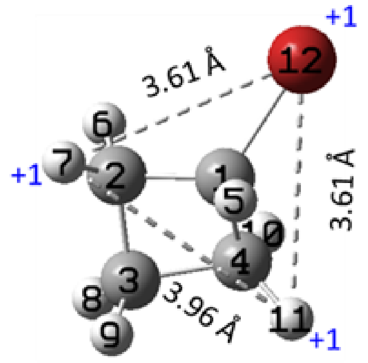
</td><td colspan="3">$CH_2^+ + C_3H_5^+ + {}^{81}Br^+$</td></tr>
<tr><td>Bonds break</td><td>$C_{(1)}$-$Br_{(12)}$<br>$C_{(2)}$-$C_{(3)}$<br>$C_{(1)}$-$C_{(2)}$</td><td>Step-1:<br>$C_{(1)}$-$Br_{(12)}$</td></tr>
<tr><td>KE($CH_2^+$)/eV</td><td>6.26</td><td></td></tr>
<tr><td>KE($C_3H_5^+$)/eV</td><td>3.60</td><td></td></tr>
<tr><td>KE($^{81}Br^+$)/eV</td><td>1.76</td><td>3.23</td></tr>
<tr><td>KER/eV</td><td>11.62</td><td></td></tr>
<tr><td colspan="4">Bromocyclo-pentane (BCpen)</td></tr>
<tr><td rowspan="11">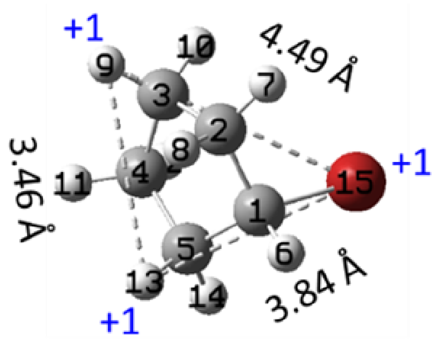
</td><td colspan="3">$CH_2^+ + C_4H_7^+ + {}^{81}Br^+$</td></tr>
<tr><td>Bonds break</td><td>$C_{(1)}$-$Br_{(15)}$<br>$C_{(3)}$-$C_{(4)}$<br>$C_{(2)}$-$C_{(3)}$</td><td>Step-1:<br>$C_{(1)}$-$Br_{(15)}$</td></tr>
<tr><td>KE($CH_2^+$)/eV</td><td>6.22</td><td></td></tr>
<tr><td>KE($C_4H_7^+$)/eV</td><td>2.96</td><td></td></tr>
<tr><td>KE($^{81}Br^+$)/eV</td><td>1.93</td><td>3.21</td></tr>
<tr><td>KER/eV</td><td>11.11</td><td></td></tr>
<tr><td>Bonds break</td><td>$C_{(1)}$-$Br_{(15)}$<br>$C_{(1)}$-$C_{(5)}$<br>$C_{(4)}$-$C_{(5)}$</td><td>Step-1:<br>$C_{(1)}$-$Br_{(15)}$</td></tr>
<tr><td>KE($CH_2^+$)/eV</td><td>6.64</td><td></td></tr>
<tr><td>KE($C_4H_7^+$)/eV</td><td>2.75</td><td></td></tr>
<tr><td>KE($^{81}Br^+$)/eV</td><td>1.72</td><td>3.21</td></tr>
<tr><td>KER/eV</td><td>11.11</td><td></td></tr>
</table>

**Table S5:** Coulomb explosion simulations for concerted and sequential breakup assuming a charge distribution that minimizes the initial Coulomb potential energy but restricts the position of the point charges to the carbon and bromine atoms. With this restriction, the simulations overestimate the experimentally observed KERs considerably.

<table>
<tr><td colspan="4">Bromocyclo-propane (BCpro)</td></tr>
<tr><td rowspan="7">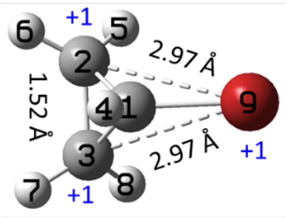
</td><td colspan="3">$CH_2^+ + C_2H_3^+ + {}^{81}Br^+$</td></tr>
<tr><td></td><td>concerted</td><td>Sequential</td></tr>
<tr><td>Bonds break</td><td>$C_{(1)}$-$Br_{(9)}$<br>$C_{(1)}$-$C_{(2)}$<br>$C_{(2)}$-$C_{(3)}$</td><td>Step-1:<br>$C_{(1)}$-$Br_{(9)}$</td></tr>
<tr><td>KE($CH_2^+$)/eV</td><td>10.72</td><td></td></tr>
<tr><td>KE($C_2H_3^+$)/eV</td><td>7.13</td><td></td></tr>
<tr><td>KE($^{81}Br^+$)/eV</td><td>1.36</td><td>3.27</td></tr>
<tr><td>KER/eV</td><td>19.21</td><td></td></tr>
<tr><td colspan="4">Bromocyclo-butane (BCbut)</td></tr>
<tr><td rowspan="6">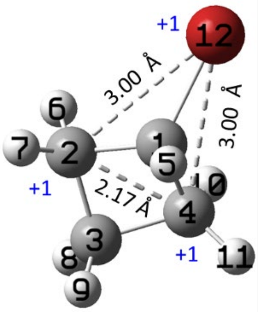
</td><td colspan="3">$CH_2^+ + C_3H_5^+ + {}^{81}Br^+$</td></tr>
<tr><td>Bonds break</td><td>$C_{(1)}$-$Br_{(12)}$<br>$C_{(2)}$-$C_{(3)}$<br>$C_{(1)}$-$C_{(2)}$</td><td>Step-1:<br>$C_{(1)}$-$Br_{(12)}$</td></tr>
<tr><td>KE($CH_2^+$)/eV</td><td>9.28</td><td></td></tr>
<tr><td>KE($C_3H_5^+$)/eV</td><td>4.90</td><td></td></tr>
<tr><td>KE($^{81}Br^+$)/eV</td><td>2.04</td><td>3.88</td></tr>
<tr><td>KER/eV</td><td>16.22</td><td></td></tr>
<tr><td colspan="4">Bromocyclo-pentane (BCpen)</td></tr>
<tr><td rowspan="11">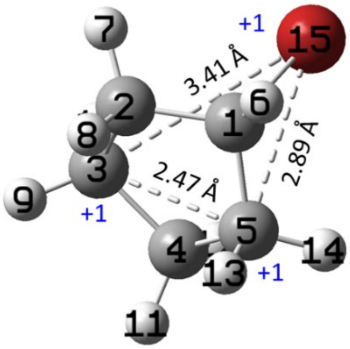
</td><td colspan="3">$CH_2^+ + C_4H_7^+ + {}^{81}Br^+$</td></tr>
<tr><td>Bonds break</td><td>$C_{(1)}$-$Br_{(15)}$<br>$C_{(3)}$-$C_{(4)}$<br>$C_{(2)}$-$C_{(3)}$</td><td>Step-1:<br>$C_{(1)}$-$Br_{(15)}$</td></tr>
<tr><td>KE($CH_2^+$)/eV</td><td>8.51</td><td></td></tr>
<tr><td>KE($C_4H_7^+$)/eV</td><td>3.96</td><td></td></tr>
<tr><td>KE($^{81}Br^+$)/eV</td><td>2.56</td><td>4.24</td></tr>
<tr><td>KER/eV</td><td>15.03</td><td></td></tr>
<tr><td>Bonds break</td><td>$C_{(1)}$-$Br_{(15)}$<br>$C_{(1)}$-$C_{(5)}$<br>$C_{(4)}$-$C_{(5)}$</td><td>Step-1:<br>$C_{(1)}$-$Br_{(15)}$</td></tr>
<tr><td>KE($CH_2^+$)/eV</td><td>9.06</td><td></td></tr>
<tr><td>KE($C_4H_7^+$)/eV</td><td>3.72</td><td></td></tr>
<tr><td>KE($^{81}Br^+$)/eV</td><td>2.25</td><td>4.24</td></tr>
<tr><td>KER/eV</td><td>15.03</td><td></td></tr>
</table>

**References:**


1. Dalitz R.H. *et al*. CXII. On the analysis of τ-meson data and the nature of the τ-meson. *The London, Edinburgh, and Dublin Philosophical Magazine and Journal of Science* **44**, 1068-1080 (1953).
2. Galster, U. *et al.* Kinematically complete final state investigations of molecular photodissociation: two- and three-body decay of laser-prepared $H_3$ $3s^2A_1'$. *Eur. Phys. J. D* **17**, 307–318 (2001).
3. Müller U. *et al*. Fragment Correlation in the Three-Body Breakup of Triatomic Hydrogen. *Phys. Rev. Lett.* **83**, 2718 (1999).
4. Ablikim U. *et al.* Identification of absolute geometries of *cis* and *trans* molecular isomers by Coulomb Explosion Imaging. *Sci Rep* **6**, 38202 (2016).
